# Correction: Measuring the impact of an interdisciplinary learning project on nursing, architecture and landscape design students’ empathy

**DOI:** 10.1371/journal.pone.0225719

**Published:** 2019-11-20

**Authors:** The PLOS ONE Staff

There are errors in the Author Contributions. The correct contributions are:

**Conceptualization:** Samantha Donnelly, Suzanne Dean

**Data curation:** Shohreh Razavy

**Formal analysis:** Shohreh Razavy

**Investigation:** Samantha Donnelly, Suzanne Dean

**Methodology: **Tracy Levett-Jones

**Project administration:** Suzanne Dean

**Supervision:** Tracy Levett-Jones

**Writing–original draft:** Samantha Donnelly

**Writing–review & editing:** Shohreh Razavy, Suzanne Dean, Tracy Levett-Jones

The second author, Suzanne Dean, is incorrectly noted as the corresponding author. The correct corresponding author is Samantha Donnelly. Dr. Donnelly’s email address is: samantha.donnelly@uts.edu.au. The publisher apologizes for these errors.
